# The Development of Gut Microbiota and Its Changes Following *C. jejuni* Infection in Broilers

**DOI:** 10.3390/vaccines11030595

**Published:** 2023-03-05

**Authors:** Walid Ghazi Al Hakeem, Keila Y. Acevedo Villanueva, Ramesh K. Selvaraj

**Affiliations:** Department of Poultry Science, College of Agricultural and Environmental Sciences, University of Georgia, Athens, GA 30602, USA

**Keywords:** broiler, microbiome, *C. jejuni*

## Abstract

The gut is home to more than millions of bacterial species. The gut bacteria coexist with the host in a symbiotic relationship that can influence the host’s metabolism, nutrition, and physiology and even module various immune functions. The commensal gut microbiota plays a crucial role in shaping the immune response and provides a continuous stimulus to maintain an activated immune system. The recent advancements in high throughput omics technologies have improved our understanding of the role of commensal bacteria in developing the immune system in chickens. Chicken meat continues to be one of the most consumed sources of protein worldwide, with the demand expected to increase significantly by the year 2050. Yet, chickens are a significant reservoir for human foodborne pathogens such as *Campylobacter jejuni.* Understanding the interaction between the commensal bacteria and *C. jejuni* is essential in developing novel technologies to decrease *C. jejuni* load in broilers. This review aims to provide current knowledge of gut microbiota development and its interaction with the immune system in broilers. Additionally, the effect of *C. jejuni* infection on the gut microbiota is addressed.

## 1. Introduction

The gastrointestinal tract of poultry species is highly populated with microorganisms that exist in a symbiotic relationship with the host [1]. These complex microbial communities benefit the host by regulating digestion and synthesizing bile acids, vitamins, and short-chain fatty acids [2]. Additionally, the gut microbiota’s competition for adhesion sites, secretion of bacteriocins, and antimicrobial peptides aid in maintaining the homeostasis of the gut [2]. The commensal gut microbiota play a crucial role in shaping the immune response, and provide a continuous stimulus to maintain an activated immune system [3]. The recent advancements in high throughput omics technologies and improved species-specific reagents have improved our understanding of the role of commensal bacteria in developing the immune system in chickens. Chicken meat continues to be one of the most consumed sources of protein worldwide, with the demand expected to increase significantly by the year 2050 [4]. Yet, chickens are a significant reservoir for human foodborne pathogens such as *Campylobacter jejuni* [5]. Understanding the interaction between the commensal bacteria and *C. jejuni* is essential in developing novel technologies to decrease the *C. jejuni* load in broilers. This review provides current knowledge of gut microbiota development and its interaction with the immune system in broilers. Additionally, the effect of *C. jejuni* infection on the gut microbiota is addressed.

## 2. Gut Microbiota and Its Development in Commercial Broilers

Domestication of chicken dates back to the Neolithic era, when humans decided to keep these birds for games and food production [6]. Birds flourished in an environment where food, water, and protection from predators were available, making their domestication easier [7]. Centuries later, the advancements in rearing methods and significant discoveries in breeding programs led to the production of the commercialized chicken we have today [7]. The intensive process of selection and breeding led to physiological and behavioral changes in domesticated chickens, which also influenced microbiota development in chickens [8]. 

The chicken gut microbiome is developed through three essential pathways: vertical, horizontal, and environmental transmission. It was thought that the development of chicken microbiota in commercially hatched broilers is achieved mainly through horizontal environmental interaction; however, recently, it was established that partial transfer of maternal microbiota into the embryo occurs during embryonic development [9]. The authors hypothesized that the microbiota present during embryonic development plays an important role in detoxifying harmful compounds generated during embryo formation. These data align with another study that reported partial passage of oviduct microbiome transmission from the hen oviduct to the embryo during egg formation [10]. Hence, these results highlight the role of vertical transmission in the chicken gut microbiome development. 

Bacterial colonization of the avian gut begins immediately after hatching and is highly affected by its surroundings [11]. During the egg-laying process, egg and feces are expelled through the cloaca, forming the eggshell’s commensal flora. The commensal flora found on the eggshell serves as a barrier against invasive pathogenic bacteria and a source for the microbiome of newly hatched chicks [12]. Previously, the eggs were in continuous contact with the hens and their nest environment, which served as a rich niche for maternal-derived microbiota [13]. However, this natural process is modified in commercial broiler production, as eggs are entirely separated from the hen. The eggs undergo several disinfection procedures before incubation to protect them from pathogenic bacteria and to increase their hatchability [14]. The egg disinfection procedure reduces eggshell commensal flora, shaping the microbiome formation in newly hatched chicks [14]. Thus, the sanitary levels at the hatcheries, the efficiency of disinfection protocols, and the different post-hatch animal handlers dictate the early gut microbiome development in newly hatched chicks. 

Day-one hatchlings arriving at the farm carry a complex microbial community in their gut. This microbial complexity is acquired during embryo development at the hatchery and during transport [15]. Several factors, including but not limited to feed, bedding material, managerial practices, environment temperature, and pathogens, influence the broiler gut microbiome. 

Feed is one of the main factors that can influence the development of the intestinal microbiome in poultry. Non-digestible feed components are inaccessible to the host and may act as a potential substrate for intestinal bacteria growth [16]. The effect of feed components is most prominent whenever corn diets are substituted with wheat, barley, or rye. Compared to corn, these cereals contain high levels of indigestible non-starch polysaccharides, which increase the digest’s viscosity, resulting in a slower passage rate [17]. These conditions favor the establishment and proliferation of *Clostridium perfringens*, potentially leading to an increased incidence of necrotic enteritis [18]. Besides feed, litter material used in chicken rearing can impact the development of gut microbial communities. The bedding material used in poultry production depends on its availability and cost in the area where poultry is raised. The type of bedding material used can influence microbial development in broilers from an early age [19]. Different pathogens can drastically impact the broiler gut microbiome. With the ban of antibiotics as growth promoters in the US and Europe, enteric pathogens are more frequently observed across broiler farms. Pathogens such as *Escherichia coli, Clostridium perfringens,* and *Salmonella* are strong colonizers that can modify the chicken [20]. Changes in the composition of the gut microbiome have been linked to intestinal and cecal dysbiosis and lower performance in infected flocks [21]. 

In summary, changes in modern poultry production created a new model of gut microbiome development. This new model, known as age-related microbiome development, does not occur when the chicks are hatched in the presence of their mother. In a study, newly hatched chicks were raised in the presence or absence of an adult hen. Twenty-four hours were needed to transfer adult-derived microbiota into the chick [22]. Future studies should focus on how to shape the broiler’s microbiome to aid the bird in achieving its full potential. 

### 2.1. Gastrointestinal Tract Microbial Composition in Commercial Broilers

#### 2.1.1. Crop 

The crop is an extension of the esophagus that works as transient storage for feed. The dry feed consumed is moistened at the crop, leading to the initiation of feed digestion. The crop function, physiology, and microbial communities are highly modified by feed composition and accessibility [23]. In the crop, the major bacteria are related to *Firmicutes,* namely facultative anaerobe *Lactobacillus* species, with *Lactobacillus reuteri, Lactobacilus salivarius,* and *Lactobacillus crispatus* being the most abundant [24]. It was noted that *Lactobacillus* species colonize the crop as early as 4 hours post-hatching [25], and they reach a concentration of 1 × 10^8^–10^9^ CFU/g [26]. Other bacterial species, including *Bifidobacterium*, *Klebsiella pneumoniae*, *Klebsiella ozaenae*, *E. coli*, *Escherichia fergusonii*, *Enterobacteraerogenes*, *Eubacterium* spp., *Pseudomonas aeruginosa*, *Micrococcus luteus*, *Staphylococcus lentus*, and *Bacillus* are also present in the crop [27]. The anaerobic conditions and stable 40 °C temperature accelerate the fermentation of sugars into organic acids, namely lactic acid and acetic acid [26]. The production of short-chain fatty acids decreases the pH of the crop, limiting the colonization of pathogenic bacteria [26]. 

#### 2.1.2. Gizzard and Proventriculus 

The presence of the gizzard and proventriculus, which constitute the true stomach in broilers, enables broilers to consume a wide variety of coarse ingredients in their diet [28]. The proventriculus is responsible for the chemical digestion of feed material by secreting hydrochloric acid and pepsinogen, while the gizzard serves as the site of mechanical digestion of feed [28]. The low pH present at the level of the gizzard acts as a barrier against pathogenic organisms. The gizzard is colonized mainly by *Lactobacillus* species, *Enterococci, Enterobacteria,* and coliforms [29]. Feed modification from a mash diet to a pellet diet can increase the presence of coliforms and anaerobic bacteria without affecting the presence of *Lactobacillus* species [30]. The high acidity of the gizzard and proventriculus limits fermentation and the production of short-chain fatty acids [2]. 

#### 2.1.3. Small Intestine 

##### Duodenum 

The short feed retention time and presence of low pH, bile, and pancreatic secretions inhibit the establishment of high bacterial density in the duodenum [31]. During the first days of the broilers, the duodenum is usually dominated by *Enterobacteriaceae* or *Clostridiacea* [32,33]. The dominance of these bacteria in broiler small intestines at a young age could be due to the ubiquitous nature of these bacteria. As the broiler matures, *Lactobacillus*, *Corynebacterium*, *Bacteroides, E. coli*, and *Shigella* colonize the duodenum [31]. 

##### Jejunum

The jejunum is the major part of the intestine responsible for lipid digestion and absorption [34], in addition to bile conversion to secondary bile acids [35]. The jejunum is characterized by low bacterial density, composed of *Lactobacillus*, *Enterococcus*, and *Bifidobacterium* [36]. The presence of microbiota is essential for the jejunum to carry out its function, as *Lactobacillus* spp., *Enterococcus* and *Staphylococcus* are equipped with bile salt hydrolase and lipase [37]. Future research will be fundamental to exploring additional bacterial species in the jejuni and identifying their role [38]. 

##### Ileum 

Undigested dietary nutrients that pass through the upper part of the gastrointestinal tract are likely substrates for the ileum microbiota. Thus, the diet has a significant influence on the ileum microbiota. *Lactobacillus* spp. dominate the microbial composition in the ileum, in addition to *Clostridiaceae*, *Streptococcus*, and *Enterococcus* spp. [39]. The fast digesta passage limits carbohydrate digestion in the ileum, resulting in lower production of short-chain fatty acids compared to the cecum [40]. 

#### 2.1.4. Ceca

**Figure 1:**
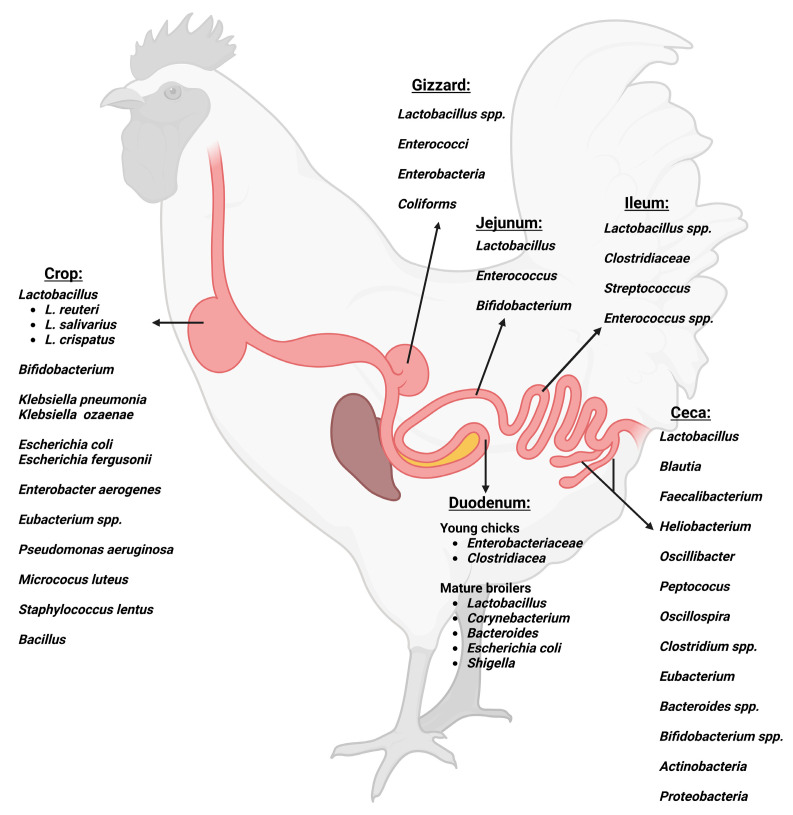
Major bacterial communities along the gastrointestinal tract of broilers. Created with BioRender.com (accessed on 29 January 2023).

The ceca are a pair of elongated chambers that arise at the junction of the ileum and the colon [41]. The presence of the highest density of microbial communities characterizes the ceca [41]. The anaerobic conditions and slow passage of digesta favor the fermentation of non-digestible starch into volatile fatty acids [42]. The bacterial density in the ceca ranges from 10^10^ to 10^11^ CFU/g of digesta [2]. Different feed formulations and nutrient availability can play a significant role in shaping the cecal microbiota. Gram-positive bacteria dominate the ceca, with the phylum *Firmicutes* being followed by *Bacteroidetes* and *Actinobacteria* [2]. The main bacterial genera identified from the *Firmicutes* phylum include *Lactobacillus*, *Blautia*, *Faecalibacterium*, *Heliobacterium*, *Oscillibacter*, *Peptococus*, *Oscillospira*, *Clostridium*, and *Eubacterium* [39]. Gram-negative bacteria represented in the *Proteobacteria* phylum are also present in the ceca, with a relative abundance ranging from 1 to 15% [43]. Despite the differences between the cecal microbial communities characterized in different studies, a core microbiome is defined in the cecum, including *Clostridium*, *Lactobacillus*, *Bacteroides*, and *Bifidobacterium*. This indicates that despite the wide variations observed in cecal microbiota, a similar core microbiome is observed across broilers [44]. Figure 1 is a schematic representation of the major bacterial communities along the gastrointestinal tract of broilers.

## 3. The Impact of the Chicken Gut Microbiome on the Avian Immune System 

The gut is home to more than 1000 bacterial species [45]. The gut bacteria coexist with the host in a symbiotic relationship that can influence the host’s metabolism, nutrition, and physiology, and even modulate various immune functions [46]. Chickens are no exception to host–microbiome interactions. However, even though chickens are well-domesticated and highly consumed animals, the gut microbiome’s influence on the immune system remains understudied. Most gut-microbiome impact research is conducted with mouse models, which has led to evidence that the gut microbiota participates in the maturation of the immune system and even alters immunological components. For example, different studies have found that the gut microbiota plays a vital role in (1) inducing the expression of α and β defensins against pathogens [47,48,49], (2) activating inflammasome pathways against pathogens [50,51], and (3) enhancing interleukin expression to clear invading pathogens [52,53]. 

Chickens can spread zoonotic diseases and harbor harmful foodborne pathogens that can impact humans [54]. Chickens are also a cheap source of protein for humans and are the second-most consumed meat worldwide [55]. Therefore, evaluating how the gut microbiome influences the chicken immune system is critical for knowing the consequences and opportunities arising from these interactions. 

### 3.1. The Chicken Gut Microbiome Impact on the Innate Immune System Components

Similar to mammals, the avian immune system comprises innate and adaptive immune systems. The innate immune system is a non-specific first line of defense against exogenous substances and invading pathogens. The innate immune system includes (1) physical barriers such as mucus, mucus membrane surfaces, and tight junctions; (2) anatomical barriers such as epithelial and phagocytic cell enzymes; (3) phagocytes such as neutrophils, monocytes, and macrophages; (4) proteins with inflammation-related functions such as the complement system and lectins; (5) granule antimicrobial peptides such as defensins; (6) different cell receptors such as Toll-like receptors; and (7) cytokine-releasing cells and inflammatory mediators such as mast cells and natural killer cells [11,12]. It is known that the gut microbiota has the capacity to condition cells to respond to both local and systemic “insults.” For example, the gut microbiota can influence intestinal macrophages to rapidly produce IL-1β in a MyD88-dependent manner to counter enteric infections [6]. For this reason, this section will explore the influence of the gut microbiome on the innate immune system components of chickens. 

#### 3.1.1. Mucin Dynamics 

A few studies with chickens have focused on the gut microbiota’s influence on the mucosal components of the innate immune system. For example, a study used germ-free chickens in comparison with conventional chickens to test how the microbial colonization of the gut influences mucin types and mucin gene expression [56]. The findings demonstrated that the lack of gut microbes results in the reduction of neutral and acidic goblet cells as well as their density, the reduced expression of Mucin 2 mRNA in the small intestine, the absence of sialylated goblet cells, and an increase in sulfated mucin. Immature goblet cells usually have more acid mucin sulfation [57]. So, these results indicate that the absence of gut microbes causes reduced mucin production and secretion, which results in a less mature small intestine mucosa. These results concur with another study that explored the effects of bacteria on intestinal goblet cell mucin production during post-hatch development in the small intestine of conventionally reared and low-bacterial-load broiler chicks [58]. In this study, the conventionally reared chicks had greater numbers of jejunum and ileal goblet cells that displayed a mucin-type of primarily sulfated acidic mucin composition, leading to an increase in sialylated sugars at four days post-hatch. These changes in mucin profiles in response to gut microbiota presence suggest that the gut microbiome has a potential role as a modulator of protective mechanisms in the intestinal mucosa. Another study by Smirnov et al. (2005) examined the effects of feeding 1-day-old chicks dietary probiotics containing the viable microorganisms *Lactobacillus acidophilus, Lactobacillus casei, Bifidobacterium bifidum,* and *Enterococcus faecium* for 14 days [59]. Results showed that the presence of the fed microorganisms resulted in a significantly enlarged goblet cell “cup” area throughout the small intestine, which significantly increased the expression of mucin mRNA, and significantly increased the levels of mucin glycoprotein in the jejunum when compared to the control. These results also corroborate that gut microbes can alter the processes of mucin biosynthesis and/or degradation. 

The exact mechanisms by which microbes affect mucin dynamics in chickens remain uncertain. Thus, further research should explore this significant research gap. Additionally, changes in mucin dynamics can impact the bird’s production performance, which is of interest to the industry and operators of small poultry enterprises. Therefore, future research should explore how the gut microbiome, individually or collectively, can influence nutrient uptake in chickens. Overall, these findings demonstrate that the gut microbiome can influence mucin dynamics in chickens. 

#### 3.1.2. Gene Expression and Macrophage-like Cells

Schokker et al. (2017) investigated the effect of modifying the normal early microbial colonization of the jejunum in day-old Cobb 500 chickens by administering antibiotics via drinking water for 24 hours [60]. Results showed that modifying the early microbial colonization had an impact on the expression of various genes that are involved in immunological processes on day 5 of age. Chickens with a disrupted microbiome had decreased expression of the genes linked to immune signaling adaptors and innate signaling, which indicated a delay in the development of cell-mediated immunity. Conversely, genes linked to cell development and intestinal barrier function were upregulated. The entire gene expression data suggest that disrupting the gut microbiome changes the developmental “priorities” of the gut. Antibiotic treatment has resulted in a shift away from cell-mediated immune development and towards strengthening the gut barrier functions. These findings suggest that gut microbiota have an essential role in strengthening gut cell-mediated immune defenses. Schokker et al. (2017) also found that on day 14 of age, the antibiotic-treated chickens had a lower number of macrophage-like cells in the jejunum tissue when compared to the control chickens [60]. These findings suggest that the downregulation of genes involved in immune processes has a direct effect on the number of macrophages. Thus, disrupting the early gut microbiome resulted in reduced or altered innate immune competence. These results indicate that the microbes that colonize the gut at an early stage in the birds’ life can significantly influence the expression of different genes and the number of macrophage-like cells. 

#### 3.1.3. Natural Killer Cells, Heterophils, and Defensins 

Natural killer cells are innate lymphoid cells that provide a rapid response to virally-infected cells [61]. A recent study found that 3-day-old broiler chickens that were inoculated with adult-derived microbiota had an increase in the percentages of intestinal IL-2Rα^+^ natural killer cells and activated natural killer cells when compared to control chickens [62]. This study demonstrates that mature microbiota can induce alterations in the presence and activation of intestinal natural killer cells, strengthening the innate immune defenses of broiler chickens. 

Heterophils are innate cells that predominate the acute inflammatory response in birds [63]. Heterophils are functionally equivalent to neutrophils in mammals [64], as they are highly phagocytic and have a broad spectrum of antimicrobial activity. Farnell et al. (2006) found that chickens that were treated with *Bacillus subtilis, Lactococcus lactis lactis,* and *Lactobacillus acidophilus* isolates exhibited a significant increase in heterophil degranulation and oxidative burst when compared with heterophils from the control birds [65]. These results suggest that the gut microbes can significantly improve the host’s immune defenses by modulating heterophil oxidative burst and degranulation in broilers.

Defensins are cationic peptides that contribute to the antimicrobial activity of phagocytes, the skin, and the mucosa [66]. Several β-defensins for the avian respiratory tract, skin, digestive tract, and urogenital tract have been described [67]. Butler et al. (2016) reported that reducing microbial exposure in birds housed in a “high hygiene” environment resulted in lower expression of duodenal avian β-defensin (AvBD) 1 and AvBD 4 at hatch and d7, with a similar trend in the ceca [68]. These findings highlight that the microbiota can significantly influence the host’s antimicrobial defenses. 

### 3.2. The Chicken Gut Microbiome Impact on the Adaptative Immune System Components

The adaptative immune system is activated in response to specific stimuli and can take up to two weeks to mount a fully developed response [61]. Even though adaptative immunity takes longer than innate immunity to mount a full response, it ultimately equips the host with “immunological memory.” Immunological memory allows the immune system to remember a specific threat and attack it faster and more efficiently. The adaptive immune system consists of specialized cellular defenses against pathogens, such as B-cells and T-cells. Mammalian research has shown that T-cells, B-cells, and immunoglobulin repertoires and responses can vary greatly depending on the gut microbes’ colonization niche and metabolic properties [69]. For example, a recent study with mice showed that immunoglobulin (Ig) D class switching happens preferentially in mucosa-associated lymphoid tissue and depends on diversified gut microbiota [70]. Conversely, the gut microbiome’s impact on the chickens’ adaptive immunity is significantly understudied. For this reason, this section will explore the influence of the gut microbiome on the adaptive immune system components of chickens.

#### 3.2.1. B-Cells and Immunoglobulins 

B-cells differentiate into plasma cells that produce different Ig isotypes depending on the type of cytokine present. For example, IgA is the most abundant antibody in mucosal surfaces and is the primary line of defense against mucosal pathogens [61,71]. In birds, notable numbers of B-cells and T-cells start to be present in the intestinal tissue at 14 days of age [63,72]. However, Kaspers et al. (2015) found that germ-free chickens (1) had underdeveloped B-cell regions and a lack of germinal centers in the cecal tonsils, (2) lacked B-cells in the lamina propria, (3) had decreased expression of genes involved in B-cell maturation and immunoglobulin class switching, and (4) were negative for IgA detection in the gut or serum up to 4 weeks of age [72]. In addition, Kaspers et al. (2015) also demonstrated that the effects could be partly rectified via mono-colonization using *Escherichia coli* Nissle or by tetra-colonization utilizing a strain of *E. coli*, *Lactobacillus*, *Enterococcus*, and *Clostridium* [72]. Another study also demonstrated that chickens exposed to maternal feces after hatching had increased IgA and IgY levels [73]. The results are consistent with other findings that indicate that germ-free chickens contain lower serum IgY compared to conventional chickens [30]. Altogether, these results demonstrate that post-hatch B-cell development and Ig levels can be influenced by the type or combination of microorganisms that colonize the chicken gut. Further research on the impact of gut microbes on the chicken immune system is needed to understand individual and collective effects of different microbiota on B-cell and Ig repertoires and responses. 

#### 3.2.2. CD4^+^ and CD8^+^ T-Cells 

The CD4^+^ T-cells are known as “helper T-cells,” and the CD8^+^ T-cells are known as “killer T-cells.” The CD4^+^ T-cells help coordinate the immune response by signaling other immune cells to fight infection, while the CD8^+^ T-cells induce cell death of intracellular pathogens by lysis or apoptosis [61]. Kaspers et al. (2015) observed that germ-free chickens also have a lack of CD4^+^ and CD8^+^ T helper cells in the gut tissues through to 4 weeks of age [72]. The results are supported by another study that found that disrupting the microbiota in day-old broilers with antibiotic treatment resulted in a numerical decrease in the number of CD4^+^ T-cells compared to birds that had non-disturbed microbial colonization [60]. Further, Meijerink et al. (2020) recently demonstrated that broiler chickens that were inoculated with adult-derived microbiota had an increase in relative numbers of intestinal cytotoxic CD8αα^+^ T cells at 14 and 21 days of age compared to control birds [62]. Altogether, findings indicate that the CD4^+^ and CD8^+^ T-cell ratio is also influenced by the gut microbiome in chickens. Future research should further explore the impact of the chicken gut microbiome on the CD4^+^/CD8^+^ T-cell ratio since it contributes to balancing the systems between inflammation and antibody production [74]. Moreover, there is a critical gap in research regarding the impact of the chicken gut microbiome on other helper T-cell subsets, particularly regulatory T (Tregs) and Th17 cell subtypes.

#### 3.2.3. T Regulatory Cells 

Chicken CD4^+^ CD25^+^ cells have suppressive properties that are equivalent to those of mammalian T regulatory cells (Tregs) [75]. The Tregs subpopulation acts to suppress the immune response, thus maintaining homeostasis and self-tolerance. A study with chickens found that disrupting the gut microbiome by administering an antibiotic cocktail in water for seven days resulted in a significant decrease in Tregs in the cecal tonsils compared to control birds [76]. The treated chickens were fed acetate and recovered the loss of Tregs in the cecal tonsils via the acetate receptor, GPR43 [76]. Other mouse studies demonstrated that the spore-forming component of indigenous intestinal microbiota, particularly clusters IV and XIVa of the genus *Clostridium*, promoted colonic Tregs [77]. Additionally, Josefowicz et al. (2012) showed that Treg polarization could also be promoted by an IL-10-dependent pathway from microbiota-derived antigen presentation by dendritic cells [78]. Overall, findings from the Lee et al. (2018) study confirm that the chicken microbiota does have an impact on Tregs [76]. However, there is a gap in the literature regarding the influence of the chicken gut microbiome on Tregs. Even though findings from studies with mammals can be used as a reference, conclusions do not necessarily apply to the avian immune system. Thus, future research should further study the impact of chicken gut microbiota on Tregs. 

#### 3.2.4. Th17 Cells 

The Th17 cell subtype plays a role in the host’s defense against extracellular pathogens and fungi, and is also linked to the development of autoimmune diseases [79]. The specific role of Th17 cells in chickens remains poorly understood [79], but a study with chickens found that the generation of Th17 cells is suppressed by Tregs [80]. It is also known that cytokine responses in different bird breeds that were orally inoculated with the bacterium *Campylobacter jejuni* result in a prominent Th17 response [81]. However, there are no studies that explore the direct effects of the chicken gut microbiota on the Th17 cell subtype. It is known that in mice, the colonization of the small intestine with a segmented filamentous bacterium is sufficient to influence the appearance of CD4^+^ T helper cells that induce Th17 cells in the lamina propria [82]. Since there are many similarities between the general immune mechanisms of mammals and chickens, we can hypothesize that the gut microbiome may modulate the host Th17 subset. However, research in this field is needed to draw specific conclusions, as there are also important differences between the mammalian and avian immune systems. Table 1 is a summary of the chicken gut microbiota effect on the innate and adaptive immune system development.

## 4. *Campylobacter jejuni* Modulation of the Avian Gut 

*C. jejuni* is a gram-negative bacterium with a sheathed flagella that is well-known for colonizing poultry [83]. *C. jejuni* is accepted to be a part of the commensal bacteria of the chicken gut. However, several experiments described the effects of *C. jejuni* infection on gut permeability, organ invasion, and proinflammatory cytokine responses, which portray it as an enteric pathogen in the chicken gut [84,85]. In humans, *Campylobacter jejuni* is the leading pathogen responsible for food poisoning in Europe and second in the US after the *Salmonella* species [86,87]. *Campylobacter jejuni* is characterized by its extensive colonization of the ceca, which could reach up to 10^9^ CFU/g [88]. Cecal rupture during processing can lead to carcass contamination. Handling and consuming contaminated poultry products leads to food poisoning [89]. No single intervention is enough to control *C. jejuni* in chickens [90]. Therefore, understanding *C. jejuni’s* modulation of the gut composition is essential to developing novel interventions to decrease *C. jejuni’s* load in broilers. 

*C. jejuni* naturally infects birds at 2–3 weeks of age, and it is rarely detected in younger chickens [91]. The early resistance to *C. jejuni* colonization in younger chickens could be attributed to maternally derived antibodies [91]. Understanding the metabolic and metal acquisition pathways of *C. jejuni* could be a starting point for understanding *C. jejuni’s* interaction with gut commensal bacteria. The genome sequence of *C. jejuni* revealed the presence of an incomplete glycolytic pathway, which could explain its inability to utilize common carbohydrates [92]. *C. jejuni* relies mainly on the citric acid cycle to generate energy, as *C. jejuni* uses several intermediates to feed this cycle. Out of the many short-chain fatty acids available in the gut, acetate [93] and lactate [94] are the only ones utilized by *C. jejuni*. To further support its growth in the gut, *C. jejuni* can use a handful of amino acids, including serine, glutamate, aspartate, asparagine, and proline [95], with the latter being only utilized in scarce conditions. 

*C. jejuni* infection alters the gut microbiome by enhancing the proliferation of certain bacteria at the expense of another [84]. As *C. jejuni* colonization reaches a high load in the infected host, it creates an environment that requires a high energy supply. This vast environment shifts the abundance of gut microbiota towards *Firmicutes* at the expense of *Proteobacteria* [96]. *Firmicutes* are gram-positive bacteria known for their ability to produce short-chain fatty acids from complex carbohydrates [97]. Therefore, *Firmicutes’* high abundance in *C. jejuni*-colonized birds supports the increased energy demand and can reduce *Proteobacteria*, namely *E. coli*, in the gut [98].

It should be noted that during microbiome establishment in broilers, *Proteobacteria* dominates in the first week and then is replaced by *Firmicutes* as the birds mature [99]. The increase in *Firmicutes* at the expense of *Proteobacteria* and the decline of maternal antibodies might explain the susceptibility of the broiler to *C. jejuni* at weeks 2–3 of age. However, this is not always the case, as the abundance and translocation of *E. coli* increase following *Campylobacter jejuni* infection [100]. *E. coli* produces a siderophore known as “enterobactin” for iron acquisition [101]. *C. jejuni* cannot produce “enterobactin”; instead, it can utilize the same siderophore produced by *E. coli* to acquire iron when resources are scarce in the host [102]. Therefore, additional experiments are required to understand the shifts related to those bacteria when *Campylobacter jejuni* colonization occurs. 

*Streptococcus* is another bacterium affected by *C. jejuni’s* presence [101]. *Streptococcus* produces lactate, a metabolic product that can be utilized by *C. jejuni* [102]. Cross-feeding between the two bacteria could explain the increase in *Streptococcus* relative abundance following *C. jejuni* infection. Differently, *Lactobacillus* spp. relative abundance is reduced following the *C. jejuni* infection [103]. *Lactobacillus* spp. dominates the gut of the broilers and plays an essential role in nutrient digestion, immune system activation, gut homeostasis, and integrity [104]. Moreover, *Lactobacillus* spp. produce organic acids and bacteriocins that inhibit the proliferation of *C. jejuni* colonization in the chicken gut [105]. The decrease in the relative abundance of *Lactobacillus* spp. can increase the colonization capacity of *C. jejuni* in infected broilers. 

A unique interaction is observed between *C. jejuni* and *C. perfringens* in the gut, as the presence of *C. jejuni* infection increases *C. perfringens* abundance [106]. *C. perfringens* can ferment complex carbohydrates [107], which generate organic acids utilized by *C. jejuni* [103]. In exchange, *C. jejuni* works as a hydrogen sink, increasing *Clostridium* spp. fermentation capacity [101]. In addition, *C. jejuni* infection increases mucin production in the gut, creating a favorable area for *Clostridium* spp. to proliferate [108]. In vitro, the coculture of *C. jejuni* and *C. perfringes* producing biofilms improved their proliferation and survivability [109]. A mechanism hypothesized to be occurring in vivo is one in which *Clostridium* spp. biofilm could be one of the mechanisms through which *C. jejuni* evades the immune system. A similar interaction is present when *Pseudomonas* spp. biofilm enhances the survivability of *Campylobacter jejuni* on different surfaces [110]. On the other hand, a reduction in *Corynebacterium* was reported following *C. jejuni* infection in broilers [101]. Non-pathogenic *Corynebacterium* is a gram-positive bacterium that plays a crucial role in maintaining mucus-associated lymphoid tissue integrity [111]. It was demonstrated that *Corynebacterium* metabolites could destroy pathogenic biofilms formed in different environments [112,113]. Therefore, their reduction might be essential for *C. jejuni*’s successful interaction with biofilm-producing bacteria and colonization of the intestine. 

**Figure 2:**
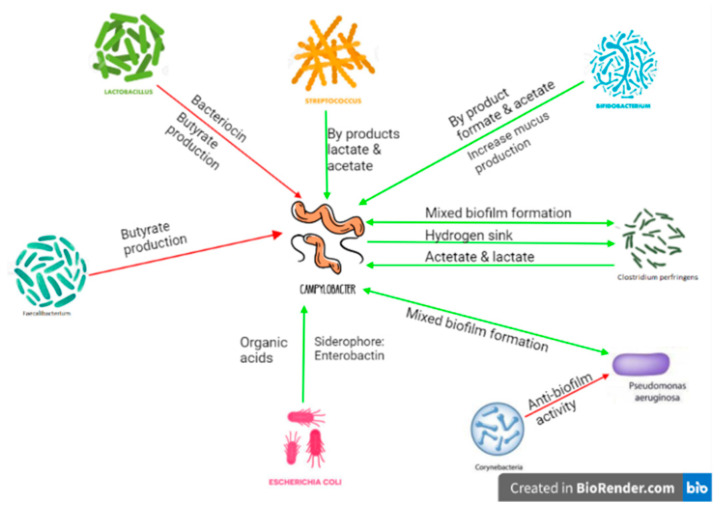
*C. jejuni* interactions with the gut microbiota. Created with BioRender.com (accessed on 29 January 2023).

Unexpected increases in certain bacteria, such as *Bifidobacterium*, following *C. jejuni* colonization have been reported [106]. *Bifidobacterium* cross-feeding with other bacteria stimulates the production of butyrate [114], which is detrimental to *C. jejuni’s* presence [115]. However, at the same time, *Bifidobacterium* can produce succinate, a carboxylic acid utilized by *Campylobacter jejuni*, when found in environments with low O_2_ levels (lower than 5%) [116]. Additionally, *Bifidobacterium* contains fucosidase, a mucin glycan-degrading enzyme, that breaks down the mucus to release fucose [117], another intermediate involved in *C. jejuni’s* citric acid cycle [118]. *C. jejuni* is accepted as an asacchrolytic organism as it lacks the ability to ferment carbohydrates. However, some studies [118,119] have revealed the presence of an L-fucose pathway in certain *C. jejuni* strains. Therefore, the presence of *Bifidobacterium* in an anaerobic environment could be beneficial for *C. jejuni*. Similarly, *C. jejuni* infection increased the abundance of *Facecalibacteruim* [106], which are other butyrate producers and vital players in reducing proinflammatory cytokines to protect the gut’s integrity [120]. Such an increase in butyrate-producing bacteria could be essential to having a tolerogenic immune reaction to *C. jejuni’s* presence by creating a downregulated environment. Figure 2 is a schematic representation of *C. jejuni’s* interactions with the gut microbiota.

## 5. Summary and Conclusions 

The latest improvements in omics technologies have increased our knowledge concerning the complex bacterial communities found in the gut. It is now established that commensal bacteria play an important role in innate and adaptive immune system development, and that foodborne pathogens such as *C. jejuni* can alter the gut microbiota. However, we are still behind in having a detailed understanding of gut microbial interactions with immune system components. Commercial poultry production changed the microbiota acquisition in young broilers, as new age-related microbiome development in broilers is now observed. Understanding the dynamics of microbial communities in broilers is essential to developing novel technologies to enhance the immune system development, and to mitigate the load of foodborne pathogens. 

## Figures and Tables

**Table 1:** Summary of the chicken gut microbiota effect on the innate and adaptive immune system development.

**Innate Immune System**		**Reference**
Mucosal components	Lack of gut microbiota can result in (1) the reduction of neutral and acidic goblet cells as well as their density, (2) the reduced expression of Mucin 2 mRNA in the small intestine, (3) the absence of sialylated goblet cells, and (4) an increase in sulfated mucin. The absence of gut microbes causes reduced mucin production and secretion resulting in a less mature small intestine mucosa.	[56]
	Conventionally reared chicks can have greater numbers of jejunum and ileal goblet cells that primarily display sulfated acidic mucin composition, leading to an increase in sialylated sugars at four days post-hatch. Changes in mucin profiles in response to the gut microbiota presence suggest that the gut microbiome has a potential role as a modulator of protective mechanisms in the intestinal mucosa.	[58]
	The presence of the fed microorganisms can result in significantly (1) enlarged goblet cell “cup” area throughout the small intestine, (2) increased expression of mucin mRNA, and (3) increased levels of mucin glycoprotein in the jejunum.	[59]
Gene expression and Macrophage-like cells	Modifying the early microbial colonization of the jejunum in day-old Cobb 500 chickens by drinking water for 24 hours can impact the expression of genes that are involved in immunological processes on day 5 of age. At day 5 of age, Cobb 500 chickens with a disrupted microbiome can have decreased expression of genes linked to immune signaling adaptors and innate signaling, indicating a delay in the development of cell-mediated immunity. Conversely, genes linked to cell development and intestinal barrier function can be upregulated. On day 14 of age, antibiotic-treated chickens can have a lower number of macrophage-like cells in the jejunum tissue. The downregulation of genes that are involved in immune processes can have a direct effect on the number of macrophages. Disrupting the early gut microbiome can result in reduced or altered innate immune competence.	[60]
Natural Killer Cells	Three-day-old broiler chickens that are inoculated with adult-derived microbiota can have an increase in the percentages of intestinal IL-2Rα^+^ natural killer cells and activated natural killer cells. The mature microbiota can induce alterations in the presence and activation of intestinal natural killer cells, strengthening the innate immune defenses of broilers.	[62]
Heterophils	Chickens that are treated with *Bacillus subtilis, Lactococcus lactis lactis,* and *Lactobacillus acidophilus* isolates can exhibit a significant increase in heterophil degranulation and oxidative burst. Gut microbes can significantly improve the host’s immune defenses by modulating heterophil oxidative burst and degranulation in broilers.	[65]
Defensins	Reducing microbial exposure in birds housed in a high hygiene environment can result in a lower expression of duodenal AvBD 1 and 4 at hatch and d7, with a similar trend in the ceca. Thus, the microbiota can significantly influence the host’s antimicrobial defenses.	[68]
**Adaptative Immune System**		**Reference**
B-cells and Immunoglobulins	Germ-free chickens can have underdeveloped B-cell regions and a lack of germinal centers in the cecal tonsils, lack B-cells in the lamina propria, decreased expression of genes involved in B-cell maturation and immunoglobulin class switching, and be negative for IgA detection in the gut or serum up to 4 weeks of age. Moreover, the effects could be partly rectified by mono-colonization using *Escherichia coli Nissle* or by tetra-colonization utilizing a strain of *E. coli*, *Lactobacillus*, *Enterococcus*, and *Clostridium.*	[72]
	Chickens exposed to maternal feces after hatching can have increased IgA and IgY levels. Conversely, germ-free chickens contain lower serum IgY levels.	[30,73]
CD4^+^ and CD8^+^ T-cells	Germ-free chickens can lack of CD4^+^ and CD8^+^ T helper cells in the gut tissues through 4 weeks of age.	[72]
	Disrupting the microbiota in day-old broilers with antibiotic treatment can result in a numerical decrease in the number of CD4^+^ T-cells.	[60]
	Broilers that are inoculated with adult-derived microbiota can have an increase in relative numbers of intestinal cytotoxic CD8αα^+^ T cells at 14 and 21 days of age.	[62]
T-regulatory Cells	Disrupting the gut microbiome by administering an antibiotic cocktail in water for seven days can result in significantly decreasing Tregs in the cecal tonsils. The treated chickens can be fed acetate and recover the loss of Tregs in the cecal tonsils via the acetate receptor, GPR43.	[76]
Th-17 Cells	A study with chickens found that the generation of Th17 cells is suppressed by Tregs.	[80]
	Cytokine responses in different bird breeds that are orally inoculated with the bacterium *Campylobacter jejuni* can result in a prominent Th17 response.	[81]

## Data Availability

Not applicable.

## References

[1] Fathima S., Shanmugasundaram R., Adams D., Selvaraj R. (2022). Gastrointestinal microbiota and their manipulation for improved growth and performance in chickens. Foods.

[2] Shang Y., Kumar S., Oakley B., Kim W.K. (2018). Chicken gut microbiota: Importance and detection technology. Front. Vet. Sci..

[3] Amoroso C., Perillo F., Strati F., Fantini M., Caprioli F., Facciotti F. (2020). The role of gut microbiota biomodulators on mucosal immunity and intestinal inflammation. Cells.

[4] Alexandratos N., Bruinsma J. (2012). World Agriculture towards 2030/2050: The 2012 Revision.

[5] Al Hakeem W.G., Fathima S., Shanmugasundaram R., Selvaraj R.K. (2022). *Campylobacter jejuni* in poultry: Pathogenesis and control strategies. Microorganisms.

[6] Price K.R., Hargis B.M., Barta J.R. (2015). From the wild red jungle fowl to domesticated chickens: Modification of eimerian-microbiome-host interactions. World’s Poult. Sci. J..

[7] Al-Nasser A., Al-Khalaifa H., Al-Saffar A., Khalil F., Albahouh M., Ragheb G., Al-Haddad A., Mashaly M. (2007). Overview of chicken taxonomy and domestication. World’s Poult. Sci. J..

[8] Mignon-Grasteau S., Narcy A., Rideau N., Chantry-Darmon C., Boscher M.-Y., Sellier N., Chabault M., Konsak-Ilievski B., Le Bihan-Duval E., Gabriel I. (2015). Impact of selection for digestive efficiency on microbiota composition in the chicken. PLoS ONE.

[9] Ding J., Dai R., Yang L., He C., Xu K., Liu S., Zhao W., Xiao L., Luo L., Zhang Y. (2017). Inheritance and establishment of gut microbiota in chickens. Front. Microbiol..

[10] Lee S., La T.-M., Lee H.-J., Choi I.-S., Song C.-S., Park S.-Y., Lee J.-B., Lee S.-W. (2019). Characterization of microbial communities in the chicken oviduct and the origin of chicken embryo gut microbiota. Sci. Rep..

[11] Cisek A., Binek M. (2014). Chicken intestinal microbiota function with a special emphasis on the role of probiotic bacteria. Pol. J. Vet. Sci..

[12] Maki J.J., Bobeck E.A., Sylte M.J., Looft T. (2020). Eggshell and environmental bacteria contribute to the intestinal microbiota of growing chickens. J. Anim. Sci. Biotechnol..

[13] Stanley D., Geier M.S., Hughes R.J., Denman S.E., Moore R.J. (2014). Highly variable microbiota development in the chicken gastrointestinal tract. PLoS ONE.

[14] Olsen R., Kudirkiene E., Thøfner I., Pors S., Karlskov-Mortensen P., Li L., Papasolomontos S., Angastiniotou C., Christensen J. (2017). Impact of egg disinfection of hatching eggs on the eggshell microbiome and bacterial load. Poult. Sci..

[15] Pedroso A., Menten J., Lambais M. (2005). The structure of bacterial community in the intestines of Newly Hatched Chicks1. J. Appl. Poult. Res.

[16] Pan D., Yu Z. (2014). Intestinal microbiome of poultry and its interaction with host and diet. Gut Microbes.

[17] Annett C., Viste J., Chirino-Trejo M., Classen H., Middleton D., Simko E. (2002). Necrotic enteritis: Effect of barley, wheat and corn diets on proliferation of *Clostridium perfringens* type A. Avian Pathol..

[18] Riddell C., Kong X.-M. (1992). The influence of diet on necrotic enteritis in broiler chickens. Avian Dis..

[19] Torok V.A., Hughes R.J., Ophel-Keller K., Ali M., MacAlpine R. (2009). Influence of different litter materials on cecal microbiota colonization in broiler chickens. Poult. Sci..

[20] Akerele G., Al Hakeem W.G., Lourenco J., Selvaraj R.K. (2022). The effect of necrotic enteritis challenge on production performance, cecal microbiome, and cecal tonsil transcriptome in broilers. Pathogens.

[21] Macdonald S.E., Nolan M.J., Harman K., Boulton K., Hume D.A., Tomley F.M., Stabler R.A., Blake D.P. (2017). Effects of Eimeria tenella infection on chicken caecal microbiome diversity, exploring variation associated with severity of pathology. PLoS ONE.

[22] Kubasova T., Kollarcikova M., Crhanova M., Karasova D., Cejkova D., Sebkova A., Matiasovicova J., Faldynova M., Pokorna A., Cizek A. (2019). Contact with adult hen affects development of caecal microbiota in newly hatched chicks. PLoS ONE.

[23] Feye K.M., Baxter M.F.A., Tellez-Isaias G., Kogut M.H., Ricke S.C. (2020). Influential factors on the composition of the conventionally raised broiler gastrointestinal microbiomes. Poult. Sci..

[24] Hilmi H.T.A., Surakka A., Apajalahti J., Saris P.E.J. (2007). Identification of the most abundant *Lactobacillus* species in the crop of 1- and 5-week-old broiler chickens. Appl. Env. Microbiol..

[25] Lev M., Briggs C. (1956). The gut flora of the chick. II. The establishment of the flora. J. Appl. Bacteriol..

[26] Rehman H.U., Vahjen W., Awad W.A., Zentek J. (2007). Indigenous bacteria and bacterial metabolic products in the gastrointestinal tract of broiler chickens. Arch. Anim. Nutr..

[27] Hinton A., Buhr R., Ingram K. (2000). Physical, chemical, and microbiological changes in the crop of broiler chickens subjected to incremental feed withdrawal. Poult. Sci..

[28] Svihus B. (2011). The gizzard: Function, influence of diet structure and effects on nutrient availability. World’s Poult. Sci. J..

[29] Gong J., Si W., Forster R.J., Huang R., Yu H., Yin Y., Yang C., Han Y. (2007). 16S rRNA gene-based analysis of mucosa-associated bacterial community and phylogeny in the chicken gastrointestinal tracts: From crops to ceca. FEMS Microbiol. Ecol..

[30] Engberg R., Hedemann M., Jensen B. (2002). The influence of grinding and pelleting of feed on the microbial composition and activity in the digestive tract of broiler chickens. Br. Poult. Sci..

[31] Xiao Y., Xiang Y., Zhou W., Chen J., Li K., Yang H. (2017). Microbial community mapping in intestinal tract of broiler chicken. Poult. Sci..

[32] Ballou A.L., Ali R.A., Mendoza M.A., Ellis J.C., Hassan H.M., Croom W.J., Koci M.D. (2016). Development of the chick microbiome: How early exposure influences future microbial diversity. Front. Vet. Sci..

[33] Jurburg S.D., Brouwer M.S.M., Ceccarelli D., van der Goot J., Jansman A.J.M., Bossers A. (2019). Patterns of community assembly in the developing chicken microbiome reveal rapid primary succession. MicrobiologyOpen.

[34] Lema I., Araújo J.R., Rolhion N., Demignot S. (2020). Jejunum: The understudied meeting place of dietary lipids and the microbiota. Biochimie.

[35] Lavelle A., Sokol H. (2020). Gut microbiota-derived metabolites as key actors in inflammatory bowel disease. Nat. Rev. Gastroenterol. Hepatol..

[36] Yang W.-Y., Lee Y., Lu H., Chou C.-H., Wang C. (2019). Analysis of gut microbiota and the effect of lauric acid against necrotic enteritis in *Clostridium perfringens* and *Eimeria* side-by-side challenge model. PLoS ONE.

[37] De Boever P., Wouters R., Verschaeve L., Berckmans P., Schoeters G., Verstraete W. (2000). Protective effect of the bile salt hydrolase-active *Lactobacillus reuteri* against bile salt cytotoxicity. Appl. Microbiol. Biotechnol..

[38] Portune K.J., Benítez-Páez A., Del Pulgar E.M.G., Cerrudo V., Sanz Y. (2017). Gut microbiota, diet, and obesity-related disorders—The good, the bad, and the future challenges. Mol. Nutr. Food Res..

[39] Lu J., Idris U., Harmon B., Hofacre C., Maurer J.J., Lee M.D. (2003). Diversity and succession of the intestinal bacterial community of the maturing broiler chicken. Appl. Environ. Microbiol..

[40] Shazali N., Loh T.C., Foo H.L., Samsudin A.A. (2019). Gut microflora and intestinal morphology changes of broiler chickens fed reducing dietary protein supplemented with lysine, methionine, and threonine in tropical environment. Rev. Bras. De Zootec..

[41] Oakley B.B., Lillehoj H.S., Kogut M.H., Kim W.K., Maurer J.J., Pedroso A., Lee M.D., Collett S.R., Johnson T.J., Cox N.A. (2014). The chicken gastrointestinal microbiome. FEMS Microbiol. Lett..

[42] Józefiak D., Rutkowski A., Martin S. (2004). Carbohydrate fermentation in the avian ceca: A review. Anim. Feed. Sci. Technol..

[43] Lee K.-C., Kil D.Y., Sul W.J. (2017). Cecal microbiome divergence of broiler chickens by sex and body weight. J. Microbiol..

[44] Ocejo M., Oporto B., Hurtado A. (2019). 16S rRNA amplicon sequencing characterization of caecal microbiome composition of broilers and free-range slow-growing chickens throughout their productive lifespan. Sci. Rep..

[45] Gilbert J.A., Blaser M.J., Caporaso J.G., Jansson J.K., Lynch S.V., Knight R. (2018). Current understanding of the human microbiome. Nat. Med..

[46] Jovel J., Dieleman L.A., Kao D., Mason A.L., Wine E. (2018). The human gut microbiome in health and disease. Metagenomics.

[47] Miani M., Le Naour J., Waeckel-Enée E., chand Verma S., Straube M., Emond P., Ryffel B., Van Endert P., Sokol H., Diana J. (2018). Gut microbiota-stimulated innate lymphoid cells support β-defensin 14 expression in pancreatic endocrine cells, preventing autoimmune diabetes. Cell Metab..

[48] Vaishnava S., Behrendt C.L., Ismail A.S., Eckmann L., Hooper L.V. (2008). Paneth cells directly sense gut commensals and maintain homeostasis at the intestinal host-microbial interface. Proc. Natl. Acad. Sci. USA.

[49] Menendez A., Willing B., Montero M., Wlodarska M., So C., Bhinder G., Vallance B., Finlay B. (2013). Bacterial stimulation of the TLR-MyD88 pathway modulates the homeostatic expression of ileal Paneth cell α-defensins. J. Innate Immun..

[50] Franchi L., Kamada N., Nakamura Y., Burberry A., Kuffa P., Suzuki S., Shaw M.H., Kim Y.-G., Núñez G. (2012). NLRC4-driven production of IL-1β discriminates between pathogenic and commensal bacteria and promotes host intestinal defense. Nat. Immunol..

[51] Levy M., Thaiss C.A., Katz M.N., Suez J., Elinav E. (2015). Inflammasomes and the Microbiota—Partners in the Preservation of Mucosal Homeostasis. Semin. Immunopathol..

[52] Nielsen M.M., Witherden D.A., Havran W.L. (2017). γδ T cells in homeostasis and host defence of epithelial barrier tissues. Nat. Rev. Immunol..

[53] Ueda Y., Kayama H., Jeon S.G., Kusu T., Isaka Y., Rakugi H., Yamamoto M., Takeda K. (2010). Commensal microbiota induce LPS hyporesponsiveness in colonic macrophages via the production of IL-10. Int. Immunol..

[54] Acevedo-Villanueva K.Y., Akerele G.O., Al Hakeem W.G., Renu S., Shanmugasundaram R., Selvaraj R.K. (2021). A novel approach against *Salmonella:* A review of polymeric nanoparticle vaccines for broilers and layers. Vaccines.

[55] Belova A.V., Smutka L., Rosochatecká E. (2012). World chicken meat market–its development and current status. Acta Univ. Agric. Et Silvic. Mendel. Brun..

[56] Cheled-Shoval S., Gamage N.W., Amit-Romach E., Forder R., Marshal J., Van Kessel A., Uni Z. (2014). Differences in intestinal mucin dynamics between germ-free and conventionally reared chickens after mannan-oligosaccharide supplementation. Poult. Sci..

[57] Turck D., Feste A.S., Lifschitz C.H. (1993). Age and diet affect the composition of porcine colonic mucins. Pediatr. Res..

[58] Forder R., Howarth G., Tivey D., Hughes R. (2007). Bacterial modulation of small intestinal goblet cells and mucin composition during early posthatch development of poultry. Poult. Sci..

[59] Smirnov A., Perez R., Amit-Romach E., Sklan D., Uni Z. (2005). Mucin dynamics and microbial populations in chicken small intestine are changed by dietary probiotic and antibiotic growth promoter supplementation. J. Nutr..

[60] Schokker D., Jansman A.J., Veninga G., de Bruin N., Vastenhouw S.A., de Bree F.M., Bossers A., Rebel J.M., Smits M.A. (2017). Perturbation of microbiota in one-day old broiler chickens with antibiotic for 24 hours negatively affects intestinal immune development. BMC Genom..

[61] Abbas A.K., Lichtman A.H., Pillai S., Baker D., Baker A. (2015). Cellular and Molecular Immunology.

[62] Meijerink N., Kers J.G., Velkers F.C., Van Haarlem D.A., Lamot D.M., De Oliveira J.E., Smidt H., Stegeman J.A., Rutten V.P., Jansen C.A. (2020). Early life inoculation with adult-derived microbiota accelerates maturation of intestinal microbiota and enhances NK cell activation in broiler chickens. Front. Vet. Sci..

[63] Davison F., Kaspers B., Schat K.A. (2008). Avian Immunology.

[64] Harmon B.G. (1998). Avian heterophils in inflammation and disease resistance. Poult. Sci..

[65] Farnell M., Donoghue A., De Los Santos F.S., Blore P., Hargis B., Tellez G., Donoghue D. (2006). Upregulation of oxidative burst and degranulation in chicken heterophils stimulated with probiotic bacteria. Poult. Sci..

[66] Vos J.B., Datson N.A., Rabe K.F., Hiemstra P.S. (2006). Exploring host-pathogen interactions at the epithelial surface: Application of transcriptomics in lung biology. Am. J. Physiol. -Lung Cell. Mol. Physiol..

[67] van Dijk A., Veldhuizen E.J., Haagsman H.P. (2008). Avian defensins. Vet. Immunol. Immunopathol..

[68] Butler V.L., Mowbray C.A., Cadwell K., Niranji S.S., Bailey R., Watson K.A., Ralph J., Hall J. (2016). Effects of rearing environment on the gut antimicrobial responses of two broiler chicken lines. Vet. Immunol. Immunopathol..

[69] Zhao Q., Elson C.O. (2018). Adaptive immune education by gut microbiota antigens. Immunology.

[70] Choi J.H., Wang K.-W., Zhang D., Zhan X., Wang T., Bu C.-H., Behrendt C.L., Zeng M., Wang Y., Misawa T. (2017). IgD class switching is initiated by microbiota and limited to mucosa-associated lymphoid tissue in mice. Proc. Natl. Acad. Sci. USA.

[71] Woof J., Russell M. (2011). Structure and function relationships in IgA. Mucosal Immunol..

[72] Kaspers B., Lettmann S., Roell S. Development of the Gut Associated Immune System. Proceedings of the 20th European Symposium on Poultry Nutrition.

[73] Zenner C., Hitch T.C., Riedel T., Wortmann E., Tiede S., Buhl E.M., Abt B., Neuhaus K., Velge P., Overmann J. (2021). Early-life immune system maturation in chickens using a synthetic community of cultured gut bacteria. Msystems.

[74] Vandaveer S., Erf G., Durdik J. (2001). Avian T helper one/two immune response balance can be shifted toward inflammation by antigen delivery to scavenger receptors. Poult. Sci..

[75] Shanmugasundaram R., Selvaraj R.K. (2011). Regulatory T cell properties of chicken CD4^+^ CD25^+^ cells. J. Immunol..

[76] Lee I.K., Gu M.J., Ko K.H., Bae S., Kim G., Jin G.-D., Kim E.B., Kong Y.-Y., Park T.S., Park B.-C. (2018). Regulation of CD4+ CD8− CD25+ and CD4+ CD8+ CD25+ T cells by gut microbiota in chicken. Sci. Rep..

[77] Atarashi K., Tanoue T., Shima T., Imaoka A., Kuwahara T., Momose Y., Cheng G., Yamasaki S., Saito T., Ohba Y. (2011). Induction of colonic regulatory T cells by indigenous Clostridium species. Science.

[78] Josefowicz S.Z., Niec R.E., Kim H.Y., Treuting P., Chinen T., Zheng Y., Umetsu D.T., Rudensky A.Y. (2012). Extrathymically generated regulatory T cells control mucosal TH2 inflammation. Nature.

[79] Kim W.H., Chaudhari A.A., Lillehoj H.S. (2019). Involvement of T cell immunity in avian coccidiosis. Front. Immunol..

[80] Kim W.H., Lillehoj H.S., Min W. (2019). Indole treatment alleviates intestinal tissue damage induced by chicken coccidiosis through activation of the aryl hydrocarbon receptor. Front. Immunol..

[81] Reid W.D.K., Close A.J., Humphrey S., Chaloner G., Lacharme-Lora L., Rothwell L., Kaiser P., Williams N.J., Humphrey T.J., Wigley P. (2016). Cytokine responses in birds challenged with the human food-borne pathogen *Campylobacter jejuni* implies a Th17 response. R. Soc. Open Sci..

[82] Ivanov I.I., Atarashi K., Manel N., Brodie E.L., Shima T., Karaoz U., Wei D., Goldfarb K.C., Santee C.A., Lynch S.V. (2009). Induction of intestinal Th17 cells by segmented filamentous bacteria. Cell.

[83] Skirrow M.B. (1994). Diseases due to *Campylobacter, Helicobacter* and related bacteria. J. Comp. Pathol..

[84] Awad W.A., Hess C., Hess M. (2018). Re-thinking the chicken–*Campylobacter jejuni* interaction: A review. Avian Pathol..

[85] Cason E.E., Al Hakeem W.G., Adams D., Shanmugasundaram R., Selvaraj R. (2022). Effects of synbiotic supplementation as an antibiotic growth promoter replacement on cecal *Campylobacter jejuni* load in broilers challenged with C. jejuni. J. Appl. Poult. Res..

[86] Kaakoush N.O., Castaño-Rodríguez N., Mitchell H.M., Man S.M. (2015). Global epidemiology of *Campylobacter* infection. Clin. Microbiol. Rev..

[87] Acevedo-Villanueva K., Akerele G., Al-Hakeem W., Adams D., Gourapura R., Selvaraj R. (2022). Immunization of broiler chickens with a killed chitosan nanoparticle *Salmonella* vaccine decreases *Salmonella* enterica serovar enteritidis load. Front. Physiol..

[88] Sahin O., Kassem I.I., Shen Z., Lin J., Rajashekara G., Zhang Q. (2015). *Campylobacter* in poultry: Ecology and potential interventions. Avian Dis..

[89] Skarp C., Hänninen M.-L., Rautelin H. (2016). Campylobacteriosis: The role of poultry meat. Clin. Microbiol. Infect..

[90] Wagenaar J.A., French N.P., Havelaar A.H. (2013). Preventing *Campylobacter* at the source: Why is it so difficult?. Clin. Infect. Dis..

[91] Pielsticker C., Glünder G., Rautenschlein S. (2012). Colonization properties of *Campylobacter jejuni* in chickens. Eur. J. Microbiol. Immunol..

[92] Parkhill J., Wren B.W., Mungall K., Ketley J.M., Churcher C., Basham D., Chillingworth T., Davies R.M., Feltwell T., Holroyd S. (2000). The genome sequence of the food-borne pathogen *Campylobacter jejuni* reveals hypervariable sequences. Nature.

[93] Wright J.A., Grant A.J., Hurd D., Harrison M., Guccione E.J., Kelly D.J., Maskell D.J. (2009). Metabolite and transcriptome analysis of *Campylobacter jejuni* in vitro growth reveals a stationary-phase physiological switch. Microbiology.

[94] Thomas M.T., Shepherd M., Poole R.K., van Vliet A.H.M., Kelly D.J., Pearson B.M. (2011). Two respiratory enzyme systems in *Campylobacter jejuni* NCTC 11168 contribute to growth on l-lactate. Environ. Microbiol..

[95] Stahl M., Butcher J., Stintzi A. (2012). Nutrient acquisition and metabolism by Campylobacter jejuni. Front. Cell. Infect. Microbiol..

[96] Awad W.A., Mann E., Dzieciol M., Hess C., Schmitz-Esser S., Wagner M., Hess M. (2016). Age-related differences in the luminal and mucosa-associated gut microbiome of broiler chickens and shifts associated with *Campylobacter jejuni* infection. Front. Cell. Infect. Microbiol..

[97] Louis P., Flint H.J. (2009). Diversity, metabolism and microbial ecology of butyrate-producing bacteria from the human large intestine. FEMS Microbiol. Lett..

[98] Parnell J., Reimer R.A. (2012). Prebiotic fiber modulation of the gut microbiota improves risk factors for obesity and the metabolic syndrome. Gut Microbes.

[99] Zhou Q., Lan F., Li X., Yan W., Sun C., Li J., Yang N., Wen C. (2021). The spatial and temporal characterization of gut microbiota in broilers. Front. Vet. Sci..

[100] Awad W.A., Dublecz F., Hess C., Dublecz K., Khayal B., Aschenbach J.R., Hess M. (2016). *Campylobacter jejuni* colonization promotes the translocation of *Escherichia coli* to extra-intestinal organs and disturbs the short-chain fatty acids profiles in the chicken gut. Poult. Sci..

[101] Kaakoush N.O., Sodhi N., Chenu J.W., Cox J.M., Riordan S.M., Mitchell H.M. (2014). The interplay between Campylobacter and Helicobacter species and other gastrointestinal microbiota of commercial broiler chickens. Gut Pathog..

[102] Belenguer A., Holtrop G., Duncan S.H., Anderson S.E., Calder A.G., Flint H.J., Lobley G.E. (2011). Rates of production and utilization of lactate by microbial communities from the human colon. FEMS Microbiol. Ecol..

[103] Connerton P.L., Richards P.J., Lafontaine G.M., O’Kane P.M., Ghaffar N., Cummings N.J., Smith D.L., Fish N.M., Connerton I.F. (2018). The effect of the timing of exposure to Campylobacter jejuni on the gut microbiome and inflammatory responses of broiler chickens. Microbiome.

[104] Dempsey E., Corr S.C. (2022). Lactobacillus spp. for gastrointestinal health: Current and future perspectives. Front. Immunol..

[105] Mortada M., Cosby D.E., Shanmugasundaram R., Selvaraj R.K. (2020). In vivo and in vitro assessment of commercial probiotic and organic acid feed additives in broilers challenged with *Campylobacter coli*. J. Appl. Poult. Res..

[106] Thibodeau A., Fravalo P., Yergeau É., Arsenault J., Lahaye L., Letellier A. (2015). Chicken caecal microbiome modifications induced by *Campylobacter jejuni* colonization and by a non-antibiotic feed additive. PLoS ONE.

[107] Fathima S., Hakeem W.G.A., Shanmugasundaram R., Selvaraj R.K. (2022). Necrotic enteritis in broiler chickens: A review on the pathogen, pathogenesis, and prevention. Microorganisms.

[108] Molnár A., Hess C., Pál L., Wágner L., Awad W., Husvéth F., Hess M., Dublecz K. (2015). Composition of diet modifies colonization dynamics of *Campylobacter jejuni* in broiler chickens. J. Appl. Microbiol..

[109] Indikova I., Humphrey T.J., Hilbert F. (2015). Survival with a Helping Hand: *Campylobacter* and Microbiota. Front. Microbiol..

[110] Ica T., Caner V., Istanbullu O., Nguyen H.D., Ahmed B., Call D.R., Beyenal H. (2012). Characterization of mono-and mixed-culture *Campylobacter jejuni* biofilms. Appl. Environ. Microbiol..

[111] Hakansson A.P., Orihuela C.J., Bogaert D. (2018). Bacterial-host interactions: Physiology and pathophysiology of respiratory infection. Physiol. Rev..

[112] Dalili D., Amini M., Faramarzi M.A., Fazeli M.R., Khoshayand M.R., Samadi N. (2015). Isolation and structural characterization of Coryxin, a novel cyclic lipopeptide from Corynebacterium xerosis NS5 having emulsifying and anti-biofilm activity. Colloids Surf. B Biointerfaces.

[113] Menberu M.A., Liu S., Cooksley C., Hayes A.J., Psaltis A.J., Wormald P.-J., Vreugde S. (2021). *Corynebacterium* accolens has antimicrobial activity against *Staphylococcus* aureus and methicillin-resistant S. aureus pathogens isolated from the sinonasal niche of chronic rhinosinusitis patients. Pathogens.

[114] Fernandez-Julia P., Commane D.M., van Sinderen D., Munoz-Munoz J. (2022). Cross-feeding interactions between human gut commensals belonging to the *Bacteroides* and *Bifidobacterium* genera when grown on dietary glycans. Microbiome Res. Rep..

[115] Van Deun K., Pasmans F., Van Immerseel F., Ducatelle R., Haesebrouck F. (2008). Butyrate protects Caco-2 cells from Campylobacter jejuni invasion and translocation. Br. J. Nutr..

[116] O’Callaghan A., van Sinderen D. (2016). *Bifidobacteria*, and Their Role as Members of the Human Gut Microbiota. Front. Microbiol..

[117] Ruas-Madiedo P., Gueimonde M., Fernández-García M., de los Reyes-Gavilán C.G., Margolles A. (2008). Mucin degradation by Bifidobacterium strains isolated from the human intestinal microbiota. Appl. Environ. Microbiol..

[118] Muraoka W.T., Zhang Q. (2011). Phenotypic and genotypic evidence for L-fucose utilization by Campylobacter jejuni. J. Bacteriol..

[119] Stahl M., Friis L.M., Nothaft H., Liu X., Li J., Szymanski C.M., Stintzi A. (2011). L-fucose utilization provides Campylobacter jejuni with a competitive advantage. Proc. Natl. Acad. Sci. USA.

[120] Lopez-Siles M., Duncan S.H., Garcia-Gil L.J., Martinez-Medina M. (2017). *Faecalibacterium prausnitzii*: From microbiology to diagnostics and prognostics. ISME J..

